# Household water, sanitation, and hygiene practices in Ghana from 1993–2022: a non-linear multivariate Poisson decomposition and traditional predictive modelling approach

**DOI:** 10.3389/fpubh.2026.1712677

**Published:** 2026-02-06

**Authors:** Bernard Afriyie Owusu, Yula Salifu, Swaray Mustapha Swithin, Joseph Lasong, Amidu Alhassan, Torjim Salifu

**Affiliations:** 1 Department of Social and Behavioural Sciences, School of Public Health, University of Ghana, Accra, Ghana; 2 Ghana Statistical Service (GSS), Accra, Ghana; 3 Department of Population, Family and Reproductive Health, School of Public Health, University of Ghana, Accra, Ghana; 4 Department of Population and Reproductive Health, School of Public Health, University for Development Studies, Tamale, Ghana; 5 Department of Adult Health, School of Nursing and Midwifery, University of Cape Coast, Cape Coast, Ghana

**Keywords:** decomposition, DHS, Ghana, predictors, sanitation and hygiene, social determinants of health, trends, water

## Abstract

**Background:**

Access to safe water, sanitation, and hygiene (WASH) remain public health and sustainable development necessities. While Ghana has made notable progress over the past three decades, persistent disparities and structural inequalities limit universal coverage. This study examined long-term trends, determinants, and rural–urban inequalities in household WASH in Ghana from 1993 to 2022 using multivariate decomposition and predictive modelling approaches.

**Methods:**

We analysed pooled data from six waves of the Ghana Demographic and Health Survey (GDHS) spanning 1993–2022 (*N* = 59,597 households). Key outcomes included access to improved water, sanitation, handwashing facilities, time to water source, and a composite WASH index. Weighted descriptive statistics, multivariable Poisson regression, and predictive modelling (AUC-ROC) assessed determinants and predictive performance. Rural–urban inequalities were quantified using a nonlinear multivariate Poisson decomposition.

**Results:**

Access to improved water rose from 56.1% in 1993 to 83.9% in 2022, while improved sanitation increased modestly to 69.1%. Handwashing facilities improved from 39.2% (2014) to 52.0% (2022), and the composite WASH index reached 18.8% in 2022. Wealth, education, female headship, and urban residence were strong predictors of improved WASH, while larger household size and more children under five reduced access. Predictive models showed good performance for improved water (AUC = 0.78) and composite WASH (AUC = 0.77). Decomposition analyses revealed persistent rural–urban gaps of 21.6 percentage points for improved water and 13.4 points for composite WASH, driven largely by wealth, education, and differential returns to socioeconomic resources. Rural households derived fewer benefits from wealth and education compared to their urban counterparts.

**Conclusion:**

Despite significant progress in WASH coverage in Ghana, inequities remain entrenched, particularly for rural and poorer households. Targeted interventions addressing structural inequalities in wealth, education, and infrastructure are essential to accelerate Ghana’s progress toward universal WASH access and Sustainable Development Goal 6.

## Background

Household water, sanitation and hygiene (WASH) are foundational determinants of population health, productivity, and human dignity. Despite substantial global progress, access to safe WASH services remains uneven. According to the World Health Organization (WHO) and the United Nations Children’s Fund (UNICEF) Joint Monitoring Program (JMP), in 2022 approximately 73% of the global population used safely managed drinking-water services, yet 2.2 billion people still lacked such services (1, 2). Globally, an estimated 3.5 billion people lacked safely managed sanitation, and 2 billion lacked basic hygiene services (2). In contrast to these global gains, sub-Saharan Africa continues to lag behind, with only 31% of the population using safely managed sanitation and 28% having access to basic hygiene services in 2020 (3). This disparity translates into a substantial health burden, with comparative risk assessments attributing over one million deaths and tens of millions of disability-adjusted life years annually to inadequate WASH, largely driven by diarrhoeal diseases (4, 5).

Ghana exemplifies both progress and persistent gaps within this broader regional context. By 2022, approximately 83.8% of the population had access to at least basic drinking-water services, defined by the JMP as water from an improved source that can be collected within a 30-min round trip (6). However, sanitation and hygiene coverage remain markedly lower. Open defecation persists, with national prevalence estimated at about 17% in 2022 (7), while only 21% of households had access to basic hand-hygiene facilities, meaning a designated place for handwashing with water and soap or cleansing agents (8, 9). Moreover, recent studies indicate that a substantial proportion of households remain exposed to faecal contamination of drinking water, even where improved sources are reported, underscoring the distinction between nominal access and effective safety (10, 11).

Multiple intersecting factors sustain these WASH deficits and position them as a critical public health and equity concern in Ghana. Rapid urbanisation and the expansion of informal settlements continue to strain existing infrastructure (12, 13). Rural residence and household poverty significantly limit access to improved sanitation and handwashing facilities (14–16), while pronounced regional disparities reflect historical underinvestment and environmental constraints (17–19). Beyond structural factors, behavioural and community-level determinants including education, media exposure, and community wealth, shape social norms around latrine use and hand hygiene and influence the uptake and sustained use of improved technologies (17, 19–21). Climate variability, flooding, and drought further threaten water reliability and quality, while affordability constraints and time burdens which are disproportionately borne by women and girls, compound vulnerability (22, 23). Collectively, these drivers perpetuate exposure to enteric pathogens, undermine child survival and nutrition, and impose avoidable economic costs on households and the health system (1, 3, 24).

These inequalities can be usefully interpreted through the WHO Social Determinants of Health (SDH) Framework, which highlights how socioeconomic position, education, gender, and place of residence shape exposure to health-promoting or health-compromising conditions (25, 26). Disparities in WASH access across households, regions, and wealth quintiles reflect structural inequities in the distribution of resources and infrastructure (27). Intermediate determinants such as community infrastructure, housing conditions, and access to information, mediate how social position translates into realised WASH outcomes (25). For instance, households with higher education and income are consistently more likely to adopt improved WASH facilities than those in deprived or informal settings (28, 29). Framing WASH inequalities within the SDH lens therefore underscores their relevance as a social justice issue requiring coordinated, intersectoral responses.

Although nationally representative surveys conducted since the 1990s permit analysis of long-term WASH trends in Ghana (30–32), important evidence gaps remain. First, many studies rely on descriptive analyses or linear and binary models that are poorly suited to count-based WASH outcomes and non-linear socio-demographic gradients. Second, decomposition analyses that distinguish changes due to population composition (e.g., education or urbanisation) from changes in associations over time remain scarce, particularly within non-linear multivariate frameworks. Third, predictive modelling is often conducted independently of decomposition approaches, limiting its utility for scenario planning and policy targeting.

To address these gaps, this study analyses household WASH practices in Ghana using data from repeated nationally representative surveys conducted between 1993 and 2022. A non-linear multivariate Poisson decomposition approach is employed because it is better suited than linear methods for modelling count-type WASH indicators and capturing non-proportional changes across population subgroups. By decomposing observed changes into compositional and coefficient components and generating out-of-sample predictions under alternative scenarios, the study provides a nuanced and policy-relevant understanding of how structural and behavioural factors jointly shape WASH access over time. While the analysis is limited to available survey measures and cannot capture all dimensions of service quality or functionality, the findings offer evidence to inform equitable targeting and accelerate progress toward Sustainable Development Goal 6.2 on sanitation and hygiene.

## Methods

### Study design and data source

This study pooled data from six rounds of the Ghana Demographic and Health Surveys (GDHS) conducted in 1993, 1998, 2003, 2008, 2014, and 2022. The GDHS is part of the global DHS Program, which provides nationally representative data on health, demographic, WASH and socioeconomic indicators. The GDHS employed a repeated cross-sectional study design using a stratified two-stage cluster sampling based on the most recent Ghana Population and Housing Census data at the time of each survey.

Enumeration areas (EAs) were selected with probability proportional to size, followed by systematic random sampling of households within EAs. Detailed methodology and survey implementation procedures have been described elsewhere.^1^

Pooling across survey years resulted in an analytical sample of 59,597 households with complete information on at least one WASH indicator. All analyses accounted for sampling weights, clustering at the primary sampling unit (PSU) level, and stratification to preserve national representativeness.

### Variable measurement

#### Outcome variables: WASH indicators

The primary outcome variables were constructed using the 2021 WHO/UNICEF Joint Monitoring Program (JMP) definitions for water, sanitation, and hygiene (1). All variables were coded as binary (1 = improved, 0 = unimproved) and then combined into a broader composite measure. Also, DHS WASH variables were harmonised across survey rounds using WHO/UNICEF JMP definitions. Differences in variable names and response categories were reconciled through recoding into standardized indicators, and surveys lacking required components were excluded from relevant analyses.

1. Improved water source.

Derived from the variable source of drinking water. Households were coded as *improved* if their source was piped water, borehole/tubewell, public tap, protected well, protected spring, or rainwater. Sources such as unprotected wells, surface water, tanker trucks, and other unspecified categories were coded as unimproved.

1. Improved sanitation facility.

Derived from the variable type of toilet facility. Improved sanitation was defined as flush/pour flush toilets, ventilated improved pit latrines, pit latrines with slabs, and composting toilets. Pit latrines without slabs, hanging toilets, bucket latrines, and shared/open defecation facilities were considered unimproved.

1. Water access within 30 min.

Constructed from the variable time to access drinking water measuring round-trip collection time. Households were coded as improved if collection time was ≤30 min, or if the source was on premises. Trips exceeding 30 min or implausible values were coded as unimproved.

1. Basic handwashing facility.

Derived from the variables: place for handwashing and availability of water and soap. A household was considered to have basic facilities if a designated place was observed and both water and soap were present. All other cases, including “do not know” responses, were coded as otherwise.

1. Composite WASH access.

A household was coded as having comprehensive WASH access only if it met all four criteria simultaneously: improved water, improved sanitation, ≤30 min access time, and basic handwashing availability.

Although the JMP framework provides a more granular classification of WASH services, reducing indicators to binary measures was a methodological decision driven by three considerations. First, not all DHS survey rounds consistently capture the information required to distinguish safely managed from basic or limited services, particularly for water quality testing, excreta disposal, and continuity of service. Second, pooling multiple survey rounds over nearly three decades necessitated a harmonised classification that could be reconstructed uniformly across time, which is more reliably achieved using binary improved versus unimproved indicators. Third, the study’s analytic objectives, which were non-linear decomposition and predictive modelling require sufficient sample sizes within categories, which would be compromised by sparse cells if full JMP ladders were retained. While this approach may mask important gradients in service quality, it allows robust estimation of long-term trends and inequalities in access, consistent with prior DHS-based WASH studies (33–35).

#### Independent variables

Several household and individual-level covariates were included, based on theoretical relevance and previous studies on WASH inequalities (33–35).

- Household composition: number of household members, number of children under five.
- Head of household characteristics:
  - Age: recoded into <25 years vs. ≥25 years.
  - Sex: male = 0, female = 1.
  - Marital status: collapsed into *never married*, *currently married/cohabiting*, and *formerly married*.
- Socioeconomic status:
  - Wealth quintile was grouped into poorest, poorer, middle, richer and richest.
  - Education was categorized as *no education, primary, secondary, and higher*+.
  - Mass media exposure was generated from radio and TV, coded as access = 1 if either was owned, otherwise 0.
- Housing characteristics:
  - Cooking fuel: *clean/improved* (electricity, liquified petroleum gas/natural gas, biogas = 1) vs. *polluting/unimproved* (otherwise = 0).
  - Shared toilet use: recoded as *no* (0) vs. *yes* (1).
  - Number of households sharing a toilet recoded into 2–4, 5–9, 10+, and do not know.
  - Toilet location coded as dwelling, yard/plot and elsewhere.
- Geographic factors: residence (urban = 0, rural = 1) and survey year (recoded into categories; 1993, 1998, 2003, 2008, 2014, 2022).

### Handling of missing data and indicator availability

A systematic approach was used to address missingness:

- Listwise deletion: All multivariable models were estimated using complete-case analysis, with Stata’s survey procedures automatically excluding cases with missing values. Missingness was below 10% for most covariates.
- Indicator availability across years: Some indicators (notably handwashing facilities) were only collected in 2014 and 2022. For these, analyses were restricted to relevant survey years to preserve data validity rather than imputing missing values. This explains varying sample sizes across WASH indicators.
- The categorical variable for survey year was initially considered for inclusion as a fixed effect in all models to account for secular trends. However, exploratory analyses revealed high collinearity between survey year and certain covariates, particularly socioeconomic status and sanitation variables, as well as structural missingness where some WASH indicators were only collected in specific rounds (handwashing facilities in 2014 and 2022). Including survey year in these models led to unstable estimates and convergence issues. Consequently, survey year was omitted from affected models to ensure statistical robustness and interpretability, while being retained in models where it did not compromise model stability. This decision reflects a balance between methodological rigor and the need to preserve valid inference from available data.
- Do not know/invalid codes: Implausible responses and “do not know” categories were recoded to missing to avoid bias.

### Statistical analysis

All analyses were conducted in Stata 18/MP.

1. Descriptive statistics: Weighted proportions for categorical variables and weighted means for continuous variables were estimated. Trends in WASH indicators were presented graphically across survey years.
2. Regression models:
  - Multivariable Poisson regression with robust variance estimators was used instead of logistic regression because of the relatively high prevalence of outcomes, producing adjusted prevalence rate ratios (APR) with 95% confidence intervals. Variables were simultaneously modelled in all regressions.
  - Models adjusted for clustering, stratification, and survey weights (svyset).
3. Traditional single-level models were estimated to account for fixed effects estimations across models. All the regression models were presented in forest plots.
4. Decomposition analysis: Non-linear multivariate Poisson decomposition (using mvdcmp) was employed to partition observed rural–urban inequalities in WASH access into differences explained by characteristics (endowments) versus effects (coefficients). All decomposition models by residence were presented in forest plots and bar charts.
5. Model fit and diagnostics: Goodness-of-fit was assessed using log-likelihood, Akaike information criterion (AIC), and Bayesian information criterion (BIC). Multicollinearity was evaluated using generalized variance inflation factor (GVIF), with values <10 considered acceptable.
6. Predictive validity: Receiver operating characteristic (ROC) curves and area under the curve (AUC) were calculated for binary outcomes, with AUC ≥0.70 indicating acceptable model discrimination.

### Ethical considerations

The GDHS obtained ethical approval from the Ghana Health Service Ethics Review Committee and the ICF Institutional Review Board. All households/participants provided informed consent prior to interview. This secondary analysis used anonymized DHS data, which are publicly available with authorization from the DHS Program, and thus did not require additional ethical approval.

## Results

### Weighted sample distribution across survey years

**Figure 1:**
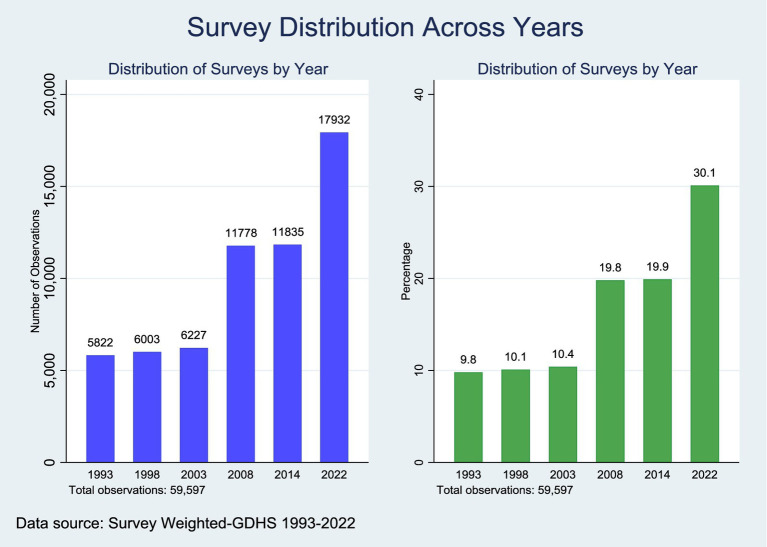
Sample estimated across survey years.

Figure 1 presents the distribution of the weighted sample across six survey waves of the Ghana Demographic and Health Survey (GDHS) conducted between 1993 and 2022. The 2022 survey represented the largest share of the pooled weighted sample (30.1%, *n* = 17,932), surveys from 2008 (19.8%, *n* = 11,778) and 2014 (19.9%, *n* = 11,835) contributed approximately equal proportions, while earlier surveys from 1993 (9.8%, *n* = 5,822), 1998 (10.1%, *n* = 6,003), and 2003 (10.4%, *n* = 6,227) each represent roughly one-tenth of the total weighted sample (Figure 1).

### Descriptive characteristics of study participants

Table 1 summarizes the weighted characteristics of 59,597 households. Most household heads were aged ≥25 years (92%) and predominantly male (64.8%). Educational attainment was skewed toward secondary education (47.3%), while 27% had no formal education. Residence was evenly distributed between urban and rural settings. Household wealth increased gradually across quintiles, with 23% in the richer group and 22.1% in the richest. On average, households had 0.61 children under five and a size of 3.7 members. Most household heads were married (62%), and over two-thirds had access to mass media (72.3%). Reliance on polluting fuels remained high (78.5%), indicating limited transition to clean energy. Sanitation conditions were constrained, as two-thirds of households shared toilets. Toilet facilities were most often located in the yard/plot (42.4%), followed by elsewhere (37.8%) and within dwellings (19.9%) (Table 1).

**Table 1:** Descriptive statistics of study participants; weighted sample of 59,597.

Variables	Weighted sample	Weighted percentage
Age of household head^a^		
Less than 25 years	4,757	8.0
25 years and above	54,838	92.0
Sex of household head		
Male	38,600	64.8
Female	20,997	35.2
Educational status of household head^a^		
No education	16,069	27.0
Primary	9,665	16.2
Secondary	28,161	47.3
Tertiary	5,678	9.5
Residence		
Urban	29,516	49.5
Rural	30,081	50.5
Household wealth status^a^		
Poorest	7,178	15.0
Poorer	8,777	18.4
Middle	10,269	21.5
Richer	10,982	23.0
Richest	10,566	22.1
Number of children under 5 years	Mean ± SD	0.609 ± 0.866
Household size	Mean ± SD	3.696 ± 2.492
Marital status of household head^a^		
Never married	7,756	14.5
Currently married	33,072	62.0
Formerly married	12,514	23.5
Household mass media access^a^		
No access	16,479	27.7
Access	43,106	72.3
Household cooking fuel use^a^		
Polluting (unimproved)	37,510	78.5
Clean (improved)	10,262	21.5
Household toilet shared^a^		
Not shared (no)	13,130	33.7
Shared (yes)	25,818	66.3
Toilet location^a^		
Dwelling	2,872	19.9
Yard/plot	6,128	42.4
Elsewhere	5,462	37.8

SD, standard deviation.

a Sample of less than weighted sample is due to data missingness.

### Trends in household WASH indicators in Ghana from 1993–2022

**Figure 2:**
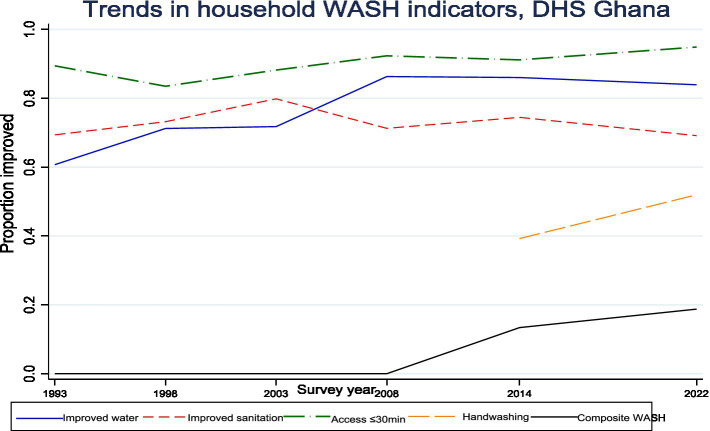
Trends in household WASH indicators in Ghana from 1993–2022.

Figure 2 depicts the evolution of household WASH indicators in Ghana over three decades. Access to improved water increased steadily, reaching 83.9% in 2022, with the most rapid gains recorded between 2003 and 2008. Improved sanitation followed a less consistent trajectory, with early progress offset by subsequent fluctuations, resulting in coverage of 69.1% in 2022. Time-to-water access remained consistently high, surpassing 83% from 1998 onward and rising to 94.9% in 2022, underscoring sustained reliability of water availability. Handwashing facilities, assessed in 2014 and 2022, showed marked progress, increasing from 39.2 to 52.0%. The composite WASH indicator, available from 2014, improved modestly to 18.8% in 2022 (Figure 2).

### Predictors of improved water and sanitation in Ghana

**Figure 3:**
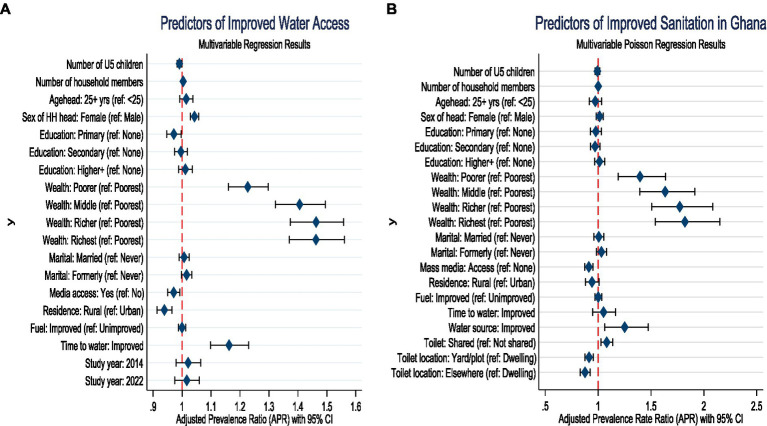
Predictors of improved water and sanitation in Ghana. **(A)** Left panel; improved water access. **(B)** Right panel; improved sanitation.

Household- and community-level characteristics were significantly associated with access to improved water. Each additional child under 5 years was associated with a slight reduction in the likelihood of improved water access (APR = 0.99, 95% CI: 0.98–0.999, *p* = 0.029). Conversely, larger household size modestly increased the probability of improved access (APR = 1.00, 95% CI: 1.00–1.01, *p* = 0.046). Female-headed households were more likely to access improved water compared to male-headed households (APR = 1.04, 95% CI: 1.03–1.06, *p* < 0.001). Relative to the poorest households, those in the poorer quintile (APR = 1.23, 95% CI: 1.16–1.30, *p* < 0.001), middle quintile (APR = 1.41, 95% CI: 1.32–1.49, *p* < 0.001), richer quintile (APR = 1.46, 95% CI: 1.37–1.56, *p* < 0.001), and richest quintile (APR = 1.46, 95% CI: 1.37–1.56, *p* < 0.001) were significantly more likely to have improved water access. In terms of informational and geographic factors, households with mass media access had lower prevalence of accessing improved water (APR = 0.97, 95% CI: 0.95–0.99, *p* = 0.008). Rural residence was also associated with reduced access compared to urban areas (APR = 0.94, 95% CI: 0.91–0.96, *p* < 0.001). Finally, households with improved time-to-water source were more likely to use improved water (APR = 1.16, 95% CI: 1.10–1.23, *p* < 0.001) (Figure 3A; left-panel).

Also, access to improved sanitation was strongly associated with socioeconomic status, household resources, and sanitation-related factors. Households in the poorer (APR = 1.40, 95% CI: 1.19–1.64, *p* < 0.001), middle (APR = 1.63, 95% CI: 1.39–1.91, *p* < 0.001), richer (APR = 1.77, 95% CI: 1.51–2.08, *p* < 0.001), and richest quintiles (APR = 1.82, 95% CI: 1.54–2.15, *p* < 0.001) were significantly more likely to report improved sanitation compared with those in the poorest households. Mass media exposure was inversely associated with improved sanitation (APR = 0.91, 95% CI: 0.87–0.95, *p* < 0.001), suggesting that households with media access were less likely to have improved sanitation facilities. Access to improved water also increased the likelihood of improved sanitation (APR = 1.25, 95% CI: 1.06–1.47, *p* = 0.007). Shared toilet facilities were positively associated with improved sanitation (APR = 1.08, 95% CI: 1.03–1.14, *p* = 0.003). By contrast, toilet location was negatively associated: facilities located in the household yard (APR = 0.91, 95% CI: 0.88–0.95, *p* < 0.001) or elsewhere (APR = 0.88, 95% CI: 0.83–0.92, *p* < 0.001) were significantly less likely to meet improved sanitation standards compared with those located inside the dwelling (Figure 3B; right-panel).

### Predictors of improved hand hygiene and time to water access

**Figure 4:**
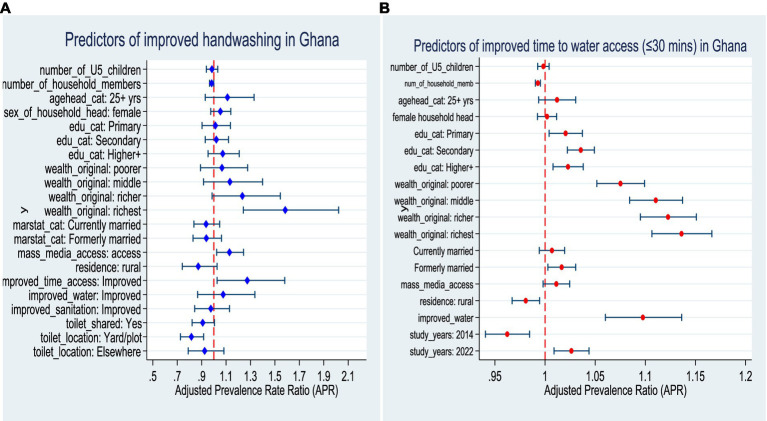
Predictors of improved hand hygiene and time to water access in Ghana. **(A)** Left panel; improved hand hygiene. **(B)** Right panel; improved time to water access.

The analysis identified several significant predictors of household access to improved handwashing facilities. Household size demonstrated a negative association, with each additional member reducing the likelihood of improved handwashing (APR = 0.98, 95% CI: 0.96–0.999, *p* = 0.042). By contrast, mass media exposure was positively associated, as households with access to media were more likely to report improved handwashing facilities (APR = 1.13, 95% CI: 1.02–1.24, *p* = 0.016). Infrastructure-related factors also played a critical role. Improved time access to water significantly increased the probability of improved handwashing (APR = 1.27, 95% CI: 1.03–1.58, *p* = 0.028). Wealth status showed a strong gradient, with the richest households exhibiting substantially higher likelihood of improved handwashing compared with the poorest (APR = 1.58, 95% CI: 1.24–2.02, *p* < 0.001). Sanitation-related variables were equally important. Toilet facilities located within household yards were significantly less likely to be associated with improved handwashing compared to those inside the dwelling (APR = 0.82, 95% CI: 0.73–0.92, *p* = 0.001) (Figure 4A; left panel).

However, the multivariable analysis revealed several significant determinants of household access to improved time to water. Larger household size was inversely associated with access, with each additional member reducing the likelihood of improved time access (APR = 0.99, 95% CI: 0.99–0.99, *p* < 0.001). Educational attainment showed a consistent positive gradient, as households headed by individuals with primary (APR = 1.02, 95% CI: 1.00–1.04, *p* = 0.014), secondary (APR = 1.04, 95% CI: 1.02–1.05, *p* < 0.001), and higher education (APR = 1.02, 95% CI: 1.01–1.04, *p* = 0.003) were significantly more likely to have improved time access. Wealth status was strongly predictive, with a progressive increase from poorer (APR = 1.08, 95% CI: 1.05–1.10, *p* < 0.001) through middle (APR = 1.11, 95% CI: 1.08–1.14, *p* < 0.001) and richer (APR = 1.12, 95% CI: 1.10–1.15, *p* < 0.001) households, reaching the highest effect among the richest (APR = 1.14, 95% CI: 1.11–1.17, *p* < 0.001). Marital history also played a role, as formerly married household heads had higher access compared to never married (APR = 1.02, 95% CI: 1.00–1.03, *p* = 0.018). Geographical disparities were observed, with rural households significantly less likely to have improved time access (APR = 0.98, 95% CI: 0.97–0.99, *p* = 0.006). Access to improved water sources was a strong enabler, with households using improved water significantly more likely to achieve improved time access (APR = 1.10, 95% CI: 1.06–1.14, *p* < 0.001). Temporal variations were also evident: access was significantly lower in 2014 compared to 2008 (APR = 0.96, 95% CI: 0.94–0.98, *p* = 0.001), but higher in 2022 (APR = 1.03, 95% CI: 1.01–1.04, *p* = 0.003) (Figure 4B; right panel).

### Predictors of improved composite household WASH in Ghana

**Figure 5:**
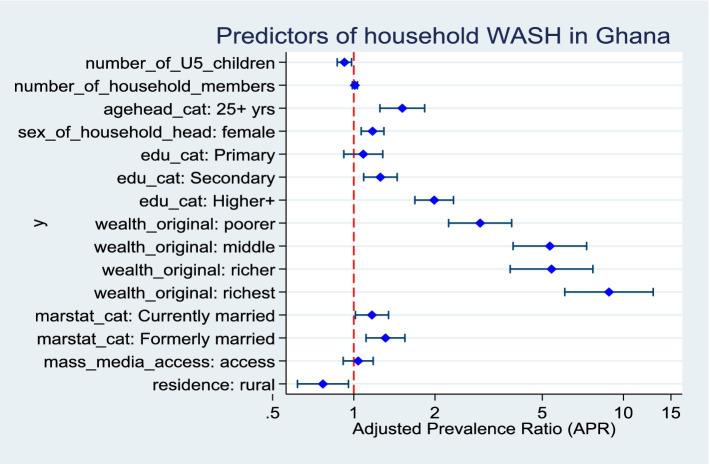
Predictors of composite household WASH in Ghana.

The analysis demonstrated several significant determinants of household access to improved WASH. Households with a greater number of under-five children were less likely to have improved WASH facilities (APR = 0.92, 95% CI: 0.87–0.98, *p* = 0.011). In contrast, household heads aged 25 years or older were substantially more likely to report improved WASH compared to younger heads (APR = 1.51, 95% CI: 1.25–1.83, *p* < 0.001). Female-headed households similarly exhibited higher access (APR = 1.17, 95% CI: 1.07–1.29, *p* = 0.001). Educational attainment exerted a pronounced influence. Households headed by individuals with secondary education (APR = 1.26, 95% CI: 1.09–1.45, *p* = 0.002) and especially those with higher education (APR = 1.99, 95% CI: 1.69–2.34, *p* < 0.001) had significantly greater access to improved WASH. Socioeconomic position showed a strong gradient: compared to the poorest, households in the poorer (APR = 2.94, 95% CI: 2.25–3.85, *p* < 0.001), middle (APR = 5.34, 95% CI: 3.90–7.30, *p* < 0.001), richer (APR = 5.41, 95% CI: 3.80–7.71, *p* < 0.001), and richest (APR = 8.84, 95% CI: 6.06–12.89, *p* < 0.001) categories were progressively more likely to access improved services. Marital status also played a role. Households headed by currently married individuals (APR = 1.17, 95% CI: 1.01–1.35, *p* = 0.031) and formerly married individuals (APR = 1.31, 95% CI: 1.11–1.55, *p* = 0.001) were more likely to have improved WASH compared to those headed by never married individuals. Finally, significant spatial inequalities were observed, as rural households were markedly less likely to report improved WASH compared to their urban counterparts (APR = 0.77, 95% CI: 0.62–0.96, *p* = 0.018) (Figure 5).

### Predictive modelling of improved WASH indicators in Ghana

**Figure 6:**
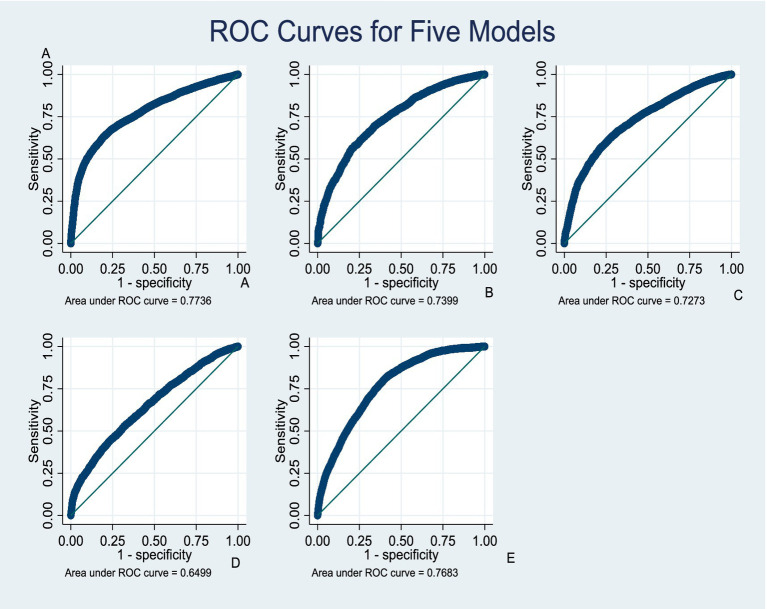
Predictive model curves (AUC-ROC); **(A)** Improved water access, **(B)** Improved sanitation, **(C)** Improved time to water access, **(D)** Improved hand hygiene, **(E)** Improved household composite WASH.

The predictive performance of the models for household WASH outcomes demonstrated varying levels of accuracy. The model for improved water access exhibited strong discriminatory capacity, with an AUC of 0.78 (95% CI: 0.77–0.78, *p* < 0.001) (Figure 6A). Similarly, the household composite WASH index performed well, with an AUC of 0.77 (95% CI: 0.76–0.78, *p* < 0.001) (Figure 6E). Improved sanitation and improved time-to-water access showed moderate discrimination, with AUCs of 0.74 (95% CI, 0.73–0.75, *p* < 0.001) (Figure 6B) and 0.73 (95% CI, 0.72–0.74, *p* < 0.001) (Figure 6C), respectively. In contrast, the model for improved handwashing facilities demonstrated relatively weaker predictive accuracy, with an AUC of 0.65 (95% CI, 0.64–0.66, *p* < 0.001) (Figure 6D).

### Multivariate Poisson decomposition analysis by place of residence across improved WASH indicators in Ghana

The Poisson decomposition analysis partitions observed changes in WASH outcomes over time into two components: changes attributable to population composition (e.g., shifts in education, wealth, or urban residence) and changes attributable to associations between these characteristics and WASH access (reflecting policy effectiveness, service delivery, and behavioural uptake). Positive percentage contributions indicate factors that facilitated improvements, whereas negative contributions indicate factors that offset or slowed progress.

### Improved water access in Ghana

**Figure 7:**
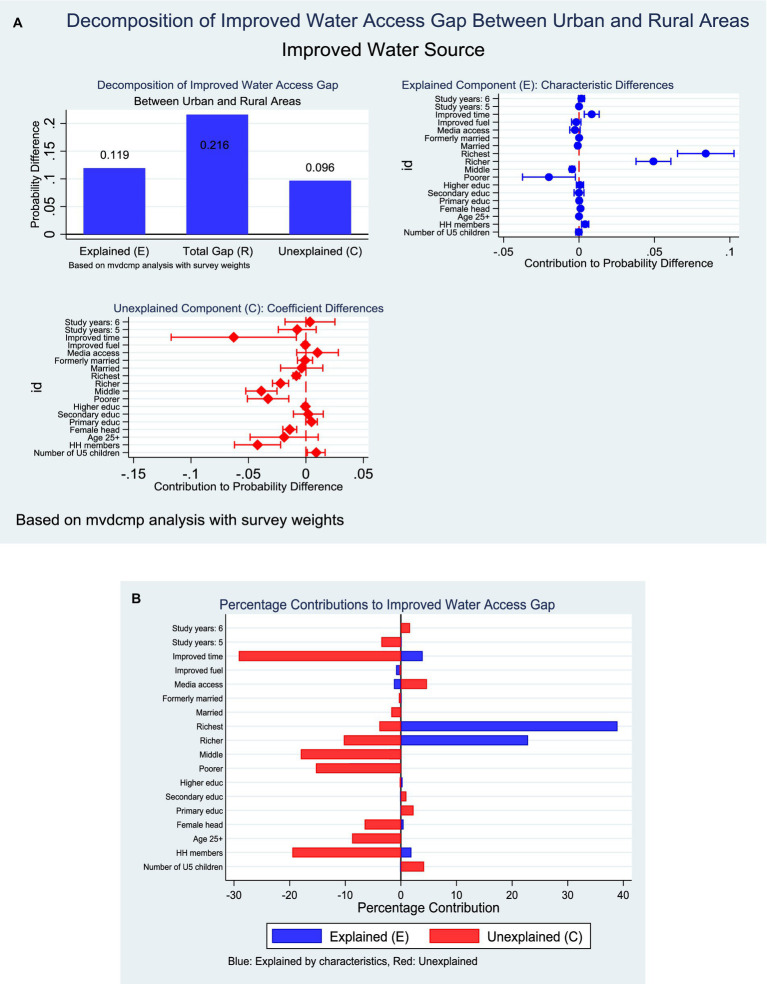
**(A)** Decomposition of improved water by residence (explained and unexplained components). **(B)** Decomposition of improved water by residence (percentage contribution).

The decomposition analysis revealed a substantial rural–urban disparity in access to improved water sources, with urban households demonstrating a markedly higher mean probability of access (95.6%) compared to rural households (67.7%). The observed gap (21.6 percentage points) was attributable to both differences in household characteristics (endowments, *E* = 55.3%) and differences in the effects of these characteristics (coefficients, *C* = 44.7%) (Figure 7A). With respect to endowments, the most important contributors were household wealth and time to water access. Belonging to the richest quintile accounted for nearly 39% of the explained gap, followed by the fourth wealth quintile (22.9%). Improved time to water access also contributed significantly (3.9%). In contrast, larger household size and female headship had smaller but statistically significant positive contributions. Regarding coefficients, several factors operated differently across residence. Notably, the negative rural effect of improved time access (−29.1%) indicated that even when rural households had better time access, its impact on improved water access was weaker compared to urban households. Similarly, higher household size (−19.5%), female headship (−6.5%), and lower wealth categories (−15.3% for second quintile and −18.0% for middle quintile) were associated with reduced returns in rural areas (Figure 7B).

### Improved sanitation in Ghana

**Figure 8:**
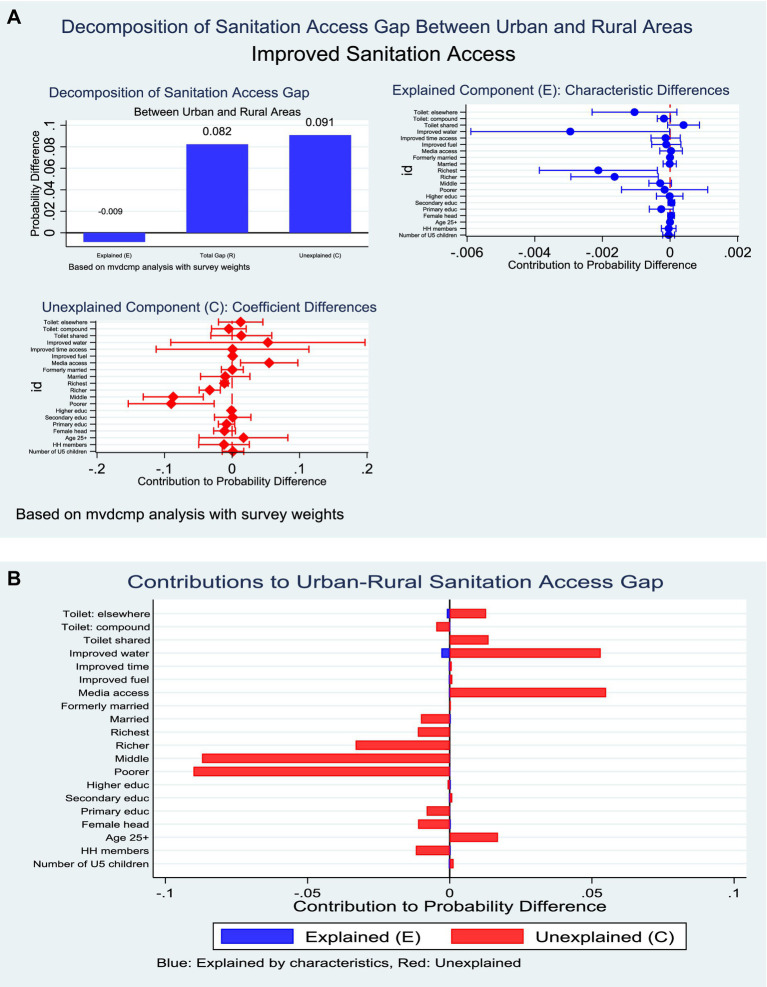
**(A)** Decomposition of improved sanitation by residence (explained and unexplained components). **(B)** Decomposition of improved sanitation by residence (percentage contribution).

The decomposition analysis of improved sanitation access showed that urban households were considerably more advantaged than their rural counterparts (84.8% vs. 59.8%). The total rural–urban gap of 8.2 percentage points was overwhelmingly explained by differences in the *effects* of household characteristics (*C* = 110.3%) rather than by differences in their distribution (*E* = −10.3%). This indicates that rural households derive fewer benefits from comparable socioeconomic and infrastructural resources. Within the endowment component, only wealth emerged as a significant driver. Belonging to the richest and fourth wealth quintiles was negatively associated with rural sanitation access (−2.6% and −2.0%, respectively), reflecting a distributional shortfall of high-wealth households in rural areas. In contrast, other compositional factors, including education, marital status, and household size, did not contribute significantly (Figure 8A).

The coefficient component revealed a more striking pattern. Differences in the effects of household wealth accounted for the largest share of inequality: rural households in the second (−109.6%), middle (−105.9%), fourth (−40.2%), and richest quintiles (−13.6%) experienced substantially diminished returns to wealth in sanitation access compared with urban households. Moreover, mass media exposure operated more strongly in urban settings, with rural households showing weaker gains (66.8%) (Figure 8B).

### Improved time to water access in Ghana

**Figure 9:**
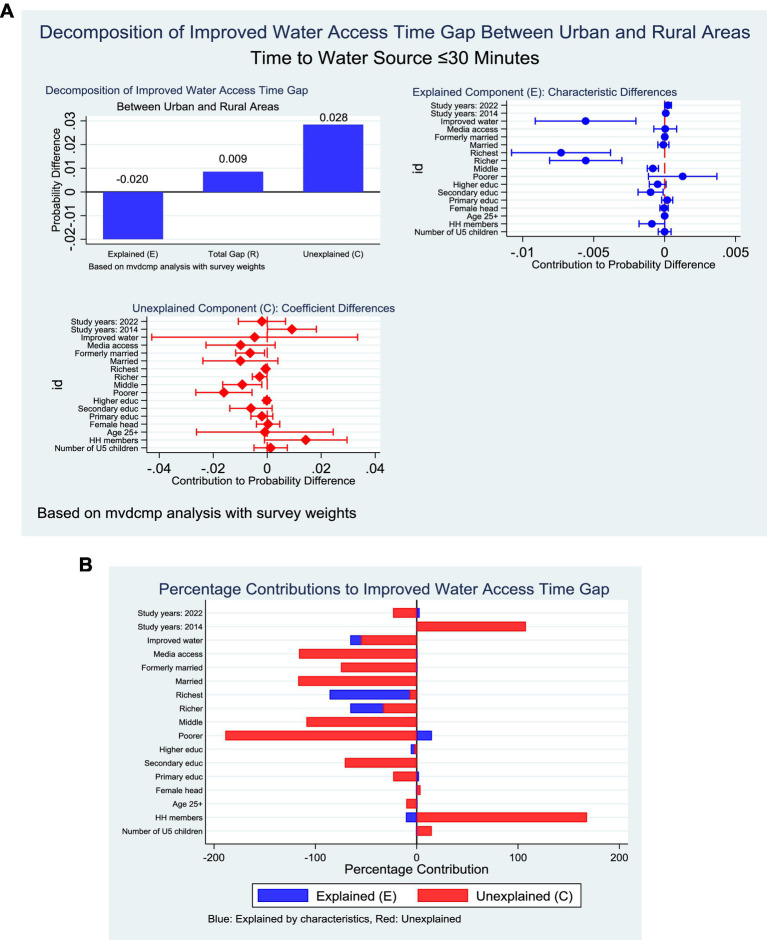
**(A)** Decomposition of improved time to water by residence (explained and unexplained components). **(B)** Decomposition of improved time to water by residence (percentage contribution).

The nonlinear multivariate decomposition analysis indicated that the rural–urban gap in improved time access (8.7 percentage points) could be partitioned into significant contributions from both endowments and coefficients. Differences in endowments contributed negatively (−0.020, *p* < 0.001), meaning that the compositional characteristics of urban households would have further widened the disparity if they had been distributed as in rural households. In contrast, differences in coefficients contributed positively (0.028, *p* < 0.001), reflecting that the structural effects of observed characteristics operated more strongly in favour of rural households. The residual unexplained component was small but significant (0.009, *p* = 0.015). At the variable level, wealth status emerged as the strongest contributor. Within the endowment effect, higher placement in the fourth and fifth quintiles contributed substantially to widening the disparity in favour of rural households (−65.5% and −85.9%, respectively; both *p* < 0.001). Improved water access also exerted a significant negative contribution (−65.6%, *p* = 0.002). In contrast, household size and education showed modest contributions, with larger households slightly disadvantaging urban households (−10.6%, *p* = 0.047), while increasing education contributed marginally positively (2.8%, *p* = 0.050) (Figure 9A).

For the coefficient effect, the differential impact of wealth across quintiles was again critical, with the second, third, and fourth quintiles significantly explaining the rural advantage (−188.8, −108.7%, and −33.0%, respectively). Marital status also contributed through coefficients, particularly among those currently married (−74.6%, *p* = 0.020). By contrast, differences in the structural effect of increasing education favoured urban households, narrowing the disparity (107.7%, *p* = 0.047) (Figure 9B).

### Improved hand hygiene in Ghana

**Figure 10:**
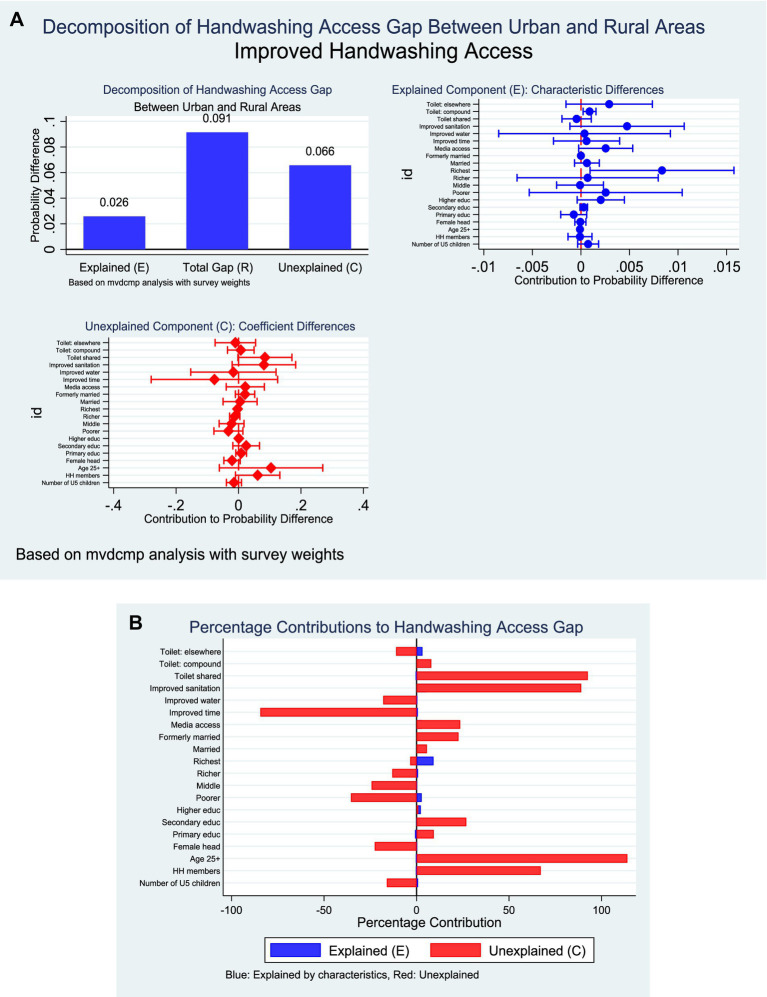
**(A)** Decomposition of improved hand hygiene by residence (explained and unexplained components). **(B)** Decomposition of improved hand hygiene by residence (percentage contribution).

The decomposition analysis revealed a substantial rural–urban disparity in access to improved handwashing facilities, with urban households having a mean probability of 55.6% compared to 38.0% among rural households. This gap of 17.6 percentage points was largely explained by both differences in endowments and differences in coefficients. Approximately 28.2% of the disparity was attributable to compositional differences (*E*), while 71.8% was explained by differences in the effects of covariates (*C*) (Figure 10A).

Within the endowment component, household wealth and toilet location emerged as significant contributors. Belonging to the richest wealth quintile accounted for 9.1% of the disparity (*p* = 0.027), while having a toilet facility within the household compound explained an additional 1.0% (*p* = 0.008). Other factors such as mass media access, improved sanitation, and education contributed modestly but were not statistically significant. Regarding the coefficient component, most contributions were not statistically significant; however, suggestive patterns were observed. Household size exerted a positive though marginal effect (6.1 percentage points, *p* = 0.092), while toilet sharing nearly reached significance (8.5 percentage points, *p* = 0.055), both indicating differences in the way these factors operated across rural and urban settings (Figure 10B).

### Improved composite household WASH

**Figure 11:**
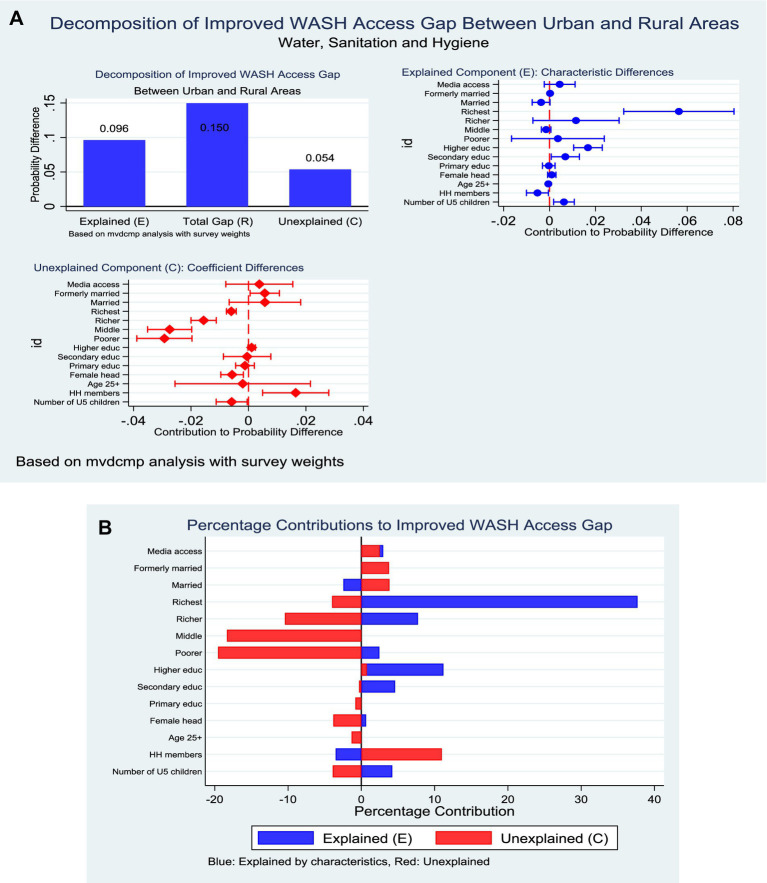
**(A)** Decomposition of improved household WASH by residence (explained and unexplained components). **(B)** Decomposition of improved household WASH by residence (percentage contribution).

The decomposition analysis demonstrated a marked rural–urban inequality in comprehensive household WASH coverage, with urban households showing a mean access level of 18.8% compared to only 5.4% among their rural counterparts. This gap of 13.4 percentage points was partitioned into 64.2% attributable to differences in characteristics (endowments) and 35.8% explained by differences in coefficients (returns to characteristics). Within the endowment component, household wealth, education, and marital status emerged as critical contributors. Belonging to the richest wealth quintile accounted for the largest share, explaining 37.7% of the disparity (*p* < 0.001). Higher education also contributed significantly, with tertiary attainment explaining 11.2% (*p* < 0.001) and secondary education adding 4.6% (*p* = 0.027). Marital status differences, particularly among formerly married household heads, contributed modestly (0.2%, *p* = 0.001). Conversely, household size had a negative contribution, reducing the explained gap by 3.5% (*p* = 0.032) (Figure 11A).

On the coefficient side, the rural–urban gap was strongly shaped by differential returns to household wealth, which together accounted for over half of the explained effect. The middle, richer, and richest wealth quintiles contributed significantly to the negative direction, reflecting weaker returns to wealth among rural households (−19.6, −18.3, and −10.4% respectively; all *p* < 0.001). Other significant contributors included household size, which explained 11.0% of the disparity (*p* = 0.005), and sex of household head, where female-headed households narrowed the gap by 3.8% (*p* = 0.005). Marital status also played a role, with formerly married heads contributing 3.8% (*p* = 0.029) (Figure 11B).

## Discussion

Access to improved drinking water in Ghana increased steadily, reaching 83.9% in 2022, with the most rapid gains occurring between 2003 and 2008, when coverage rose by approximately 12 percentage points. This period coincided with intensified public and donor investments in rural boreholes, small-town piped systems, and urban network expansion, reflecting a strong policy and financing focus on water supply infrastructure. Similarly, access to water within a 30-min round trip improved markedly, with 94.9% of households in 2022 meeting this threshold, substantially reducing time burdens, particularly for women and children (1, 36).

In contrast, progress in improved sanitation was slower and more volatile, reaching 69.1% in 2022, with intermittent stagnation and reversals across survey waves. These fluctuations reflect structural financing and delivery challenges that disproportionately affect sanitation relative to water. Unlike water supply, sanitation in Ghana relies heavily on household-level investment, limited public subsidies, and fragmented donor support, resulting in uneven uptake. Public expenditure on sanitation infrastructure has historically been lower than for water, and sanitation programs are often short-term, project-based, and donor-dependent, undermining sustainability. Similar sanitation stagnation despite water gains has been documented in Nigeria, Ethiopia, and Tanzania, where sanitation financing, enforcement, and maintenance remain weak despite expanded water coverage (1, 24).

Behavioural and socio-cultural barriers further explain why sanitation lags behind water. Persistent norms around open defecation, low prioritization of latrine construction relative to other household needs, tenancy arrangements in urban informal settlements, and weak enforcement of sanitation by-laws limit sustained adoption of improved facilities. These barriers affect sanitation more than water because water access delivers immediate and visible benefits, whereas sanitation requires sustained behaviour change, private investment, and collective compliance to achieve community-wide benefits.

Access to basic handwashing facilities also lags behind water improvements. Although coverage increased from 39.2% in 2014 to 52.0% in 2022, nearly half of households still lack a designated place with both water and soap. This gap reflects affordability constraints, inconsistent availability of soap, and the absence of hygiene infrastructure in sanitation facilities, particularly in poorer and rural households. Similar hygiene lags have been reported across sub-Saharan Africa, where hygiene promotion has not kept pace with water infrastructure development (37).

As a result, the composite WASH indicator, which requires simultaneous access to improved water, sanitation, short collection time, and handwashing facilities, remained low at 18.8% in 2022. This underscores that gains in single components, especially water, do not automatically translate into integrated WASH access. For Ghana, these patterns imply that future WASH strategies must move beyond water-centric investments to rebalance financing toward sanitation and hygiene, strengthen enforcement and behaviour-change interventions, and integrate social determinants of health into WASH planning to achieve equitable and sustained progress.

### Predictors of improved household WASH

Access to improved WASH in Ghana is shaped by household, community, and structural determinants, which can be conceptualized using the social determinants of health (SDH) framework. This framework highlights how socioeconomic position, education, gender roles, and place of residence shape health behaviours and access to essential services (25, 26).

Wealth consistently shaped outcomes across all domains, with households in higher quintiles significantly more likely to have improved water, sanitation, handwashing facilities, and composite WASH access as confirmed in other studies (14–16, 28, 29). This reflects entrenched inequalities in affordability of services and infrastructural investments, a trend echoed across sub-Saharan Africa (1, 24). Educational attainment of household heads further enhanced access, as households headed by individuals with secondary or higher education were far more likely to achieve improved WASH, suggesting that literacy and health awareness facilitate adoption of safe water and sanitation practices (38).

Female-headed households also had better access to improved water and composite WASH, consistent with evidence that women’s autonomy and prioritization of household health resources translate into improved environmental conditions (39). Gender- and household-level theories, including gender and intra-household bargaining theory, further elucidate why female headship often improves WASH outcomes: women frequently allocate household resources toward health-promoting goods, prioritizing sanitation, hygiene, and water quality due to caregiving roles and heightened risk perception.

Household size and composition showed mixed associations: while larger households increased the likelihood of improved water, they were less likely to maintain improved handwashing facilities, indicating resource strain and behavioural challenges when more individuals share limited facilities. The presence of children under five further reduced the probability of improved water and composite WASH, reflecting competing demands on household resources and possible prioritization of immediate caregiving over infrastructural improvements.

At the community level, geographic location, infrastructural proximity, and informational exposure were significant determinants of WASH access. Urban households had markedly higher access to improved water, sanitation, and composite WASH compared to rural households, underscoring persistent spatial inequalities in infrastructure distribution and investment (40). Access to improved water sources within shorter collection times strongly predicted both improved water use and handwashing facilities, highlighting the role of infrastructure availability in reducing collection burdens, particularly for women and children (14, 19).

Mass media exposure showed paradoxical effects. It improved hand hygiene practices but was inversely associated with improved water and sanitation access. This likely reflects urban–rural and infrastructural disparities, where households with better media connectivity may remain underserved in basic services, highlighting the limitations of information alone without accompanying infrastructure.

Community sanitation infrastructure also shaped outcomes, as shared facilities increased the probability of improved sanitation, whereas off-premises facilities reduced it, reflecting both accessibility and quality dimensions. These patterns emphasise that while household resources and behaviours are critical, structural inequities in infrastructure delivery and regional disparities remain central obstacles. Addressing Ghana’s WASH gaps thus requires integrated strategies that combine household empowerment with systemic community-level investments to reduce spatial inequalities and promote equitable progress toward SDG 6.

### Residence-based decomposition of WASH

Also, decomposition results reveal profound rural–urban disparities across all WASH indicators, highlighting both compositional (endowment) and structural (coefficient) drivers. For improved water access, the 21.6-percentage point gap between urban (95.6%) and rural households (67.7%) was explained almost equally by household characteristics (55.3%) and differences in their effects (44.7%). Wealth was the dominant driver, with the richest quintiles contributing nearly two-thirds of the explained gap, consistent with earlier findings that socioeconomic status is a major determinant of water access in Ghana and sub-Saharan Africa (24, 39). However, the weaker rural returns to favourable factors such as improved time-to-water access and female headship underscore structural disadvantages that limit the impact of resources in rural contexts. This aligns with regional studies showing that rural water systems are more vulnerable to infrastructural breakdowns and governance inefficiencies (29).

For sanitation, the urban advantage (84.8% vs. 59.8%) was overwhelmingly driven by coefficients (110.3%), indicating that rural households derive fewer benefits from wealth and resources compared with their urban counterparts. Even when rural households belong to higher wealth quintiles, the returns to sanitation access are substantially diminished, reflecting the infrastructural deficits and entrenched cultural practices of open defecation in rural settings (41, 42). Mass media exposure also exerted stronger effects in urban settings, where information is likely reinforced by better service availability, further widening inequalities.

For time-to-water access, the rural–urban gap (8.7 percentage points) presented a more complex picture. Endowment effects were negative, suggesting that rural compositional characteristics, if equally distributed, would have widened disparities further. Instead, rural households benefited from relatively stronger structural effects of wealth and marital status, which reduced the observed gap. This finding suggests that certain protective dynamics such as community solidarity and reliance on shared water points, may mitigate structural disadvantages in rural contexts, echoing evidence on social resilience in water collection practices (43, 44). In terms of hygiene, the urban advantage (55.6% vs. 38.0%) was explained primarily by coefficient effects (71.8%), with wealth and toilet location driving compositional disparities. The weaker predictive power of household characteristics for hygiene is consistent with the broader literature that links handwashing uptake more to behavioural norms and social mobilisation than to wealth or infrastructure alone (45).

Finally, composite WASH coverage exhibited the starkest inequality (18.8% vs. 5.4%), with wealth and education as dominant contributors on the endowment side and diminished returns to wealth in rural areas explaining most of the coefficient effects. The findings affirm that WASH inequalities in Ghana are not only a function of resource distribution but also of systemic inequities in how those resources translate into services across rural and urban contexts. Addressing these disparities will require dual strategies: targeted infrastructural investments in underserved areas and structural reforms to enhance the returns of existing resources, supported by behavioural interventions to shift sanitation and hygiene practices. These results reinforce the need for equity-focused WASH programming, echoing global calls to “leave no one behind” in the pursuit of SDG 6 (1, 24).

### Implications for public health policy and practice

The study underscores the urgent need for equity-driven WASH strategies in Ghana. Although access to improved water has expanded significantly, sanitation and hygiene lag, and rural–urban disparities remain substantial. Policy responses must therefore prioritize reducing structural inequalities by strengthening the Ministry of Sanitation and Water Resources (MSWR)’s rural programming, supported by financing from development partners such as the World Bank, African Development Bank (AfDB), and UNICEF. The decomposition results show that wealth, education, and female headship influence WASH access, but rural households often derive fewer benefits from these characteristics, a major inequality in resources. This indicates the necessity for inclusive policies that go beyond infrastructure provision, tackling systemic barriers that limit rural returns on socioeconomic resources.

Regulatory bodies such as the Environmental Protection Agency (EPA) and district assemblies should enforce equitable service distribution and incentivise private-sector participation in rural sanitation markets. Alignment with Sustainable Development Goal (SDG) 6 requires cross-sectoral integration, adequate financing, and robust monitoring frameworks to accelerate universal access.

Findings reveal actionable opportunities for strengthening implementation at household, community, and institutional levels. The Ghana Health Service (GHS), in collaboration with the National Commission for Civic Education (NCCE) and media platforms, should recalibrate behavioural campaigns to address the paradoxical effect of mass media positively influencing handwashing but inversely associated with sanitation.

Context-specific communication, particularly through community radio and local influencers, will be critical to promote affordable sanitation and hygiene behaviours. Civil society organisations, notably the Coalition of NGOs in Water and Sanitation (CONIWAS), can facilitate community mobilisation, while social protection schemes such as LEAP (Livelihood Empowerment Against Poverty) can be leveraged to subsidise WASH access for the poorest households.

At the operational level, integrated interventions like safe water provision, improved sanitation, and hygiene promotion delivered together should be prioritized rather than pursued separately. Local government authorities, supported by traditional leaders, can ensure culturally sensitive adoption, while academia and research institutions provide continuous evaluation to guide adaptive programming.

### Strengths and limitations

A key strength of this study lies in its use of nationally representative data spanning three decades (1993–2022), allowing for robust analysis of long-term WASH trends in Ghana. The large, pooled sample size enhanced statistical power and generalizability of the findings. Applying a nonlinear multivariate Poisson decomposition approach provided deeper insights into the drivers of rural–urban inequalities, distinguishing between compositional and structural factors. Additionally, predictive modelling and ROC analysis enabled rigorous assessment of model performance and future applicability of identified determinants.

Nevertheless, several limitations should be acknowledged. First, the analysis relied on cross-sectional data, which limits causal inference between predictors and WASH outcomes. Second, self-reported household characteristics may be subject to recall and social desirability biases, particularly regarding sanitation and hygiene practices. Third, some key contextual factors such as governance, community-level infrastructure, and cultural practices were not fully captured in the DHS datasets, which may have introduced residual confounding.

Fourth, handwashing data were only available in two survey rounds (2014 and 2022), limiting the ability to track long-term trends for hygiene indicators. Finally, decomposition results may be sensitive to model specification and variable measurement, and unobserved factors could still explain part of the disparities observed. Despite these limitations, the study provides comprehensive and policy-relevant evidence on WASH progress and inequities in Ghana, highlighting priority areas for targeted interventions.

## Conclusion

This study highlights substantial progress in household WASH access in Ghana over the past three decades, particularly in improved water and sanitation. However, progress in hand hygiene and comprehensive WASH coverage remains limited, with marked rural–urban disparities and persistent inequalities shaped by social determinants of health notably wealth, education, household composition, and place of residence. Rural households and the poorest quintiles consistently derived fewer benefits from comparable socioeconomic resources, underscoring structural inequities in the effectiveness of interventions. To accelerate equitable WASH improvements, policies must prioritize rural households and disadvantaged wealth groups. Investments in rural infrastructure, including piped water systems and improved sanitation facilities, are critical to narrowing gaps. Programs should integrate targeted subsidies and social protection schemes to enhance affordability for poorer households, aligning with the frameworks emphasis on improving material circumstances and social protection.

Education, especially of household heads, strongly predicted improved WASH; thus, integrating WASH messaging into adult literacy and community education campaigns could strengthen uptake. Female-headed households were more likely to achieve improved access, suggesting opportunities to leverage women’s roles in household decision-making and community-level WASH programs as a pathway to strengthen social capital and empowerment which are core elements of the SDH framework. Finally, predictive modelling confirmed the robustness of determinants such as wealth, education, and urban residence.

Policymakers should use these predictive insights to guide data-driven planning, prioritize resource allocation, and design tailored interventions that address both compositional disadvantages (low wealth, low education) and contextual disadvantages (rural residence, weak infrastructure). Framing WASH equity within the SDH framework underscore that without deliberate action to address the underlying social and structural determinants of access, composite WASH coverage by 2030 (SDG 6) will remain unattainable.

## Data Availability

Publicly available datasets were analyzed in this study. This data can be found at: https://dhsprogram.com/data/available-datasets.cfm.

## Footnotes

1
https://dhsprogram.com/data/available-datasets.cfm/
